# Significance of Viral Activity for Regulating Heterotrophic Prokaryote Community Dynamics along a Meridional Gradient of Stratification in the Northeast Atlantic Ocean

**DOI:** 10.3390/v12111293

**Published:** 2020-11-12

**Authors:** Kristina D. A. Mojica, Corina P. D. Brussaard

**Affiliations:** 1 Department of Biological Oceanography, Royal Netherlands Institute for Sea Research (NIOZ), P.O. Box 59, 1790 AB Den Burg, Texel, The Netherlands; Corina.Brussaard@nioz.nl; 2 Division of Marine Science, School of Ocean Science and Engineering, The University of Southern Mississippi, Stennis Space Center, MS 39529, USA

**Keywords:** bacterial production, marine viruses, mortality, lytic infection, lysogeny, protozoan grazing, carbon cycling, HNA, LNA

## Abstract

How microbial populations interact influences the availability and flux of organic carbon in the ocean. Understanding how these interactions vary over broad spatial scales is therefore a fundamental aim of microbial oceanography. In this study, we assessed variations in the abundances, production, virus and grazing induced mortality of heterotrophic prokaryotes during summer along a meridional gradient in stratification in the North Atlantic Ocean. Heterotrophic prokaryote abundance and activity varied with phytoplankton biomass, while the relative distribution of prokaryotic subpopulations (ratio of high nucleic acid fluorescent (HNA) and low nucleic acid fluorescent (LNA) cells) was significantly correlated to phytoplankton mortality mode (i.e., viral lysis to grazing rate ratio). Virus-mediate morality was the primary loss process regulating the heterotrophic prokaryotic communities (average 55% of the total mortality), which may be attributed to the strong top-down regulation of the bacterivorous protozoans. Host availability, encounter rate, and HNA:LNA were important factors regulating viral dynamics. Conversely, the abundance and activity of bacterivorous protozoans were largely regulated by temperature and turbulence. The ratio of total microbial mediated mortality to total available prokaryote carbon reveals that over the latitudinal gradient the heterotrophic prokaryote community gradually moved from a near steady state system regulated by high turnover in subtropical region to net heterotrophic production in the temperate region.

## 1. Introduction

Marine heterotrophic prokaryotes mediate fundamental processes of biogeochemical cycles and are an integral component of planktonic food webs. Studying the factors driving their spatiotemporal variation is essential for a better understanding of the functioning of marine ecosystems. Flow cytometric analysis of prokaryotes routinely reveals two distinct clusters of prokaryotic cells [1,2], referred to as high nucleic acid fluorescent (HNA) and low nucleic acid fluorescent (LNA) prokaryote populations [3]. While these subpopulations appear to be ubiquitous in aquatic systems [4,5], their relative distributions can vary widely across environmental conditions and geographic regions [4,6,7,8]. To date, studies have largely focused on the role that disparity in the metabolic potential of LNA and HNA cells plays in driving spatiotemporal variations in the relative proportion of these prokaryote subpopulations and their activity within and among aquatic systems [1,9,10,11,12,13]. Microbial abundance and activity, however, are a consequence of the net balance between growth and mortality.

Mortality processes regulate biomass, community composition and size structure, and elemental cycling of microbial communities [14,15,16,17]. Grazing by phagotrophic protozoans (particularly bacterivorous nanoflagellates) and viral infection are the primary top-down processes regulating heterotrophic prokaryote populations in aquatic environments [15,18]. These mortality agents have distinct influences on food web dynamics and ecosystem processes, as they affect the flow of carbon and energy in substantially different ways. Bacterivory is an integral pathway by which dissolved organic carbon (DOC) is reincorporated into the food web via bacterial biomass. The lysis of microbes by viral activity, on the other hand, redirects energy and matter towards microbial reprocessing, enhancing bacterial respiration and the regeneration of nutrients, which are in turn utilized by the primary producers [19,20,21]. Grazers and viruses also affect the structure of heterotrophic prokaryote communities in very different ways. Grazing modifies prokaryote biomass and size-structure through prey size preference [15,22,23], although some protozoans, particularly flagellates, exhibit partialities for particular prey species or morphotypes [23,24]. Viruses, on the other hand, are host-specific and consequently are powerful drivers of biodiversity [16,25,26].

Several studies have explored the potential for mortality processes to regulate the share of prokaryotic subpopulations in natural systems. The proportion of HNA cells has been shown to increase in response to reductions in bacterivores, consistent with size- and activity-selective behavior of flagellates [9,27,28], however, preferential grazing on LNA cells has also been reported [13]. Similarly, viral abundance and the fraction of HNA cells correlated significantly across different aquatic systems [29]. The same study also found a significantly higher abundance of intracellular virus particles in HNA sorted cells within each system. More recently, the abundance and lytic production of two different virus subpopulations were correlated with the cellular production and abundance of HNA and LNA subpopulations in the North Atlantic [30]. Until now, however, the scarcity of concurrent measurements of protozoan grazing and viral mortality within prokaryotic communities have limited our ability directly compare the magnitude by which these mortality sources affect and regulate prokaryotic subpopulations. 

Here we assess the prokaryotic abundance and production, relative proportion of LNA and HNA cells in the prokaryote community, viral reproductive strategy (lytic vs. lysogenic), and viral induced mortality relative to grazing of the prokaryotes over a latitudinal gradient across the North Atlantic Ocean during summer stratification. We employ multivariate analysis to identify key factors driving the geographical variation in abundance, growth, and mortality of subprokaryotic populations. Specifically, we assess the following hypotheses: (H1) the heterotrophic prokaryote community production and relative abundance of LNA and HNA cells will vary according to the concentration of phytoplankton carbon in the water column, which is strongly influenced by water column stratification [31]. Due to their reliance on their host to provide the energy and metabolic machinery required for replication (H2) viral abundance, viral replication mode (i.e., lytic and lysogenic) and the production of viruses through lytic infection will vary with heterotrophic prokaryote community production and relative abundance of LNA and HNA cells. Wherein viral abundance and lytic production is positively associated with heterotrophic prokaryote community production and active members of the community. Bacterivorous protozoans, on the other hand, may be directly affected by stratification through alterations in temperature and turbulence [32,33,34,35,36], and consequently be governed by distinct physicochemical forcing. Therefore, we hypothesize that (H3) the abundance and grazing activity of bacterivorous protozoans will be strongly tied to temperature and turbulence in the water column rather than characteristics of their prey community.

## 2. Materials and Methods 

### 2.1. Sampling and Physicochemical Parameters

**Figure 1:**
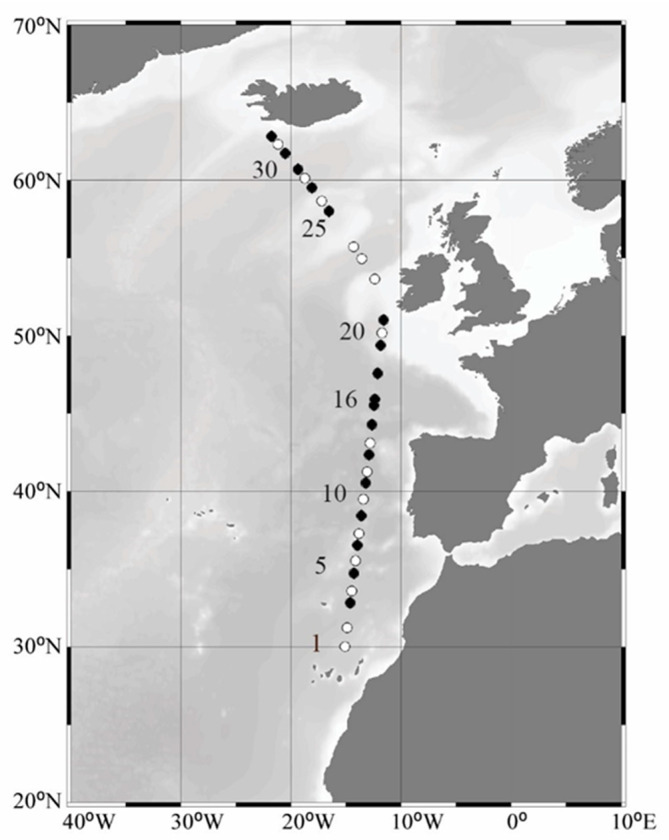
North-south gradient across the Northeast Atlantic Ocean. Bathymetric map depicting stations sampled during the summer STRATIPHYT. Mortality assays to determine viral lysis and microzooplankton grazing rates were performed at stations indicated by black symbols. Figure was prepared using Ocean Data View version 5.2.

Thirty-two stations were sampled along a latitudinal gradient in the Northeast Atlantic Ocean during the shipboard expedition of STRATIPHYT which took place onboard of the R/V Pelagia in July–August of 2009 (Figure 1). The water column along the transect was stratified with relatively consistent and shallow mixed layer depths (MLD) ranging from 18–46 m [37]. Water samples were collected at each station prior to dawn using 24 plastic samplers (General Oceanics type Go-Flow, 10 L) mounted on an ultra-clean (trace-metal free) system consisting of a fully titanium sampler frame equipped with CTD (Seabird 9+; standard conductivity, temperature and pressure sensors) and auxiliary sensors for chlorophyll autofluorescence (Chelsea Aquatracka Mk III). Water samples were collected inside a 6 m clean container. Data from the chlorophyll autofluorescence sensor were calibrated against HPLC data according to van de Poll et al. (2013) [38]. Phytoplankton carbon (PhytoC) was estimated from phytoplankton cell counts obtained using flow cytometry [31].

Methods and data for temperature eddy diffusivity (K_T_) and dissolved inorganic nutrients have been discussed previously [31,37]. In short, K_T_ (referred to here as vertical mixing) was derived from temperature and conductivity microstructure profiles measured using a SCAMP (self-contained autonomous microprofiler), deployed at 14 stations and down to 100 m depth. For the additional stations and depths, data were interpolated using the spatial kriging function ‘krig’ executed in R using the ‘fields’ package. Brunt–Väisälä frequency (N^2^), was used to quantify the strength of stratification and was determined from CTD data processed with SBE Seabird software according to the Fofonoff adiabatic leveling method.

Samples for dissolved inorganic phosphate (PO_4_), ammonium (NH_4_), nitrate (NO_3_), and nitrite (NO_2_) were gently filtered through 0.2 µm pore size polysulfone Acrodisk filters (32 mm, Pall Corp., Port Washington, USA) and stored at −20 °C until analysis. Dissolved inorganic nutrients were analyzed onboard using a Bran + Luebbe Quaatro AutoAnalyzer for dissolved orthophosphate (PO_4_), inorganic nitrogen (nitrate + nitrite: NO_x_) and ammonium (NH_4_). Detection limits were 0.10 µM for NO_x_, 0.028 µM for PO_4_ and 0.09 µM for NH_4_. 

At each station, water samples were obtained from 3–10 depths for the enumeration of heterotrophic prokaryotes (HPA), heterotrophic nanoflagellates (HNF), and viruses (VA) and from three separate depths for heterotrophic prokaryote production (HPP), virus production (VP) and heterotrophic protozoan grazing measurements. Sampling depths for rate-based measurements were classified as: mixed layer (ML; 15 m), MID (25–85 m), which contained the deep-chlorophyll maximum (DCM) when present, and DEEP (100–225 m). The DCM (47–85 m) was defined by the presence of a subsurface peak in the vertical profile of Chl *a* autofluorescence. In addition, at four stations the ML was sampled for a second time prior to dusk (i.e., 7–2, 11–2, 17–2 and 30–2).

### 2.2. Microbial Abundances

Heterotrophic prokaryotes (bacteria and archaea) and viruses were enumerated using a Becton-Dickinson FACSCalibur flow cytometer equipped with an air-cooled Argon laser with an excitation wavelength of 488 nm (15 mW) according to Marie et al. [39], with modifications according to Mojica et al. [40]. Briefly, samples were fixed with 25% glutaraldehyde (EM-grade, Sigma-Aldrich, Zwijndrecht, the Netherlands) at a final concentration of 0.5% for 15–30 min at 4 °C, flash frozen and stored at −80 °C until analysis. Thawed samples were diluted using TE buffer, pH 8.2 (10 mM Tris-HCL, 1 mM EDTA; Roche, Mannheim, Germany). Prokaryote samples were stained in the dark at room temperature for 15 min using SYBR Green I at a final concentration of 1 × 10^−4^ of the commercial stock. Virus samples were stained by heating in the dark at 80 °C for 10 min in the presence of the nucleic acid-specific green fluorescence dye SYBR Green I at a final concentration of 0.5 × 10^−4^ of the commercial stock concentration (Life Technologies, Bleiswijk, the Netherlands). Trigger for analysis was set on green fluorescence and the obtained list-mode files were analyzed using the freeware, CYTOWIN [41]. Two distinct clusters of prokaryotic cells were distinguished based on their nucleic acid-specific green fluorescence, i.e., a high nucleic acid (HNA) and a low nucleic acid (LNA) fluorescent prokaryote population [1,2]. The contribution of Prochlorococcus to the flow cytometric signal of the HNA subpopulation was assessed using bivariate scatter plots of green versus red chlorophyll autofluorescence. Prochlorococcus was detected in the ML and DCM of the oligotrophic southern stations (<45.5 °N) comprising 11.8% and 4.7% of the total prokaryote abundance, respectively. For the enumeration of viruses, only the V1-V3 virus populations [42] were considered in this study. 

Heterotrophic nanoflagellates (HNF) were enumerated by epifluorescence microscopy. Briefly, 20 mL of seawater was fixed using 25% glutaraldehyde to a final concentration of 1% (10% working stock, Sigma Aldrich) and stained for 30 min using 4′6-diamidino-2-phenylindole dihydrochloride (DAPI) (1 mg mL^−1^, Sigma-Aldrich, Zwijndrecht, the Netherlands) at a final concentration of 2 µg mL^−1^. Samples were filtered onto 0.2 µm black polycarbonate filter (25 mm, Whatman, Maidstone, UK) and stored at −20 °C. A minimum of 75 fields and 100 HNF in were counted using a Zeiss Axiophot epifluorescence microscope equipped with BP 365, FT395 and LP397 excitation filters. HNF were distinguished by their size, presences of a flagella, and absence of a chloroplast.

### 2.3. Heterotrophic Prokaryotic Production

Heterotrophic prokaryotic production (HPP) was determined from leucine incorporation rates according to Simon and Azam [43]. Ten-milliliter seawater samples were taken in triplicate. One sample was used as a control to which 0.5 mL formaldehyde (37%; Sigma-Aldrich, Zwijndrecht, The Netherlands) was added in order to kill the prokaryotes. Thirty µL [3H] leucine (specific activity, 139 Ci mmol^−1^; Amersham, Little Chalfont, UK) was added to each sample, equivalent to 50 µCurie per vial, and incubated in the dark at in situ temperature for 2 h. Samples were then fixed with 0.5 mL formaldehyde (37%; Sigma-Aldrich, Zwijndrecht, the Netherlands) and filtered onto 0.2 µm polycarbonate filters (25 mm, Whatman, Maidstone, UK). Filters were washed twice by addition of 5% chilled trichloroacetic acid (TCA) for 5 min and then transferred to scintillation vials and stored at −80 °C until analysis. Prior to analysis, 8 mL of scintillation cocktail (Filter-Count LCS cocktail; PerkinElmer, Waltham, MS, USA) was added and left for 6 h. Samples were analyzed using a LKB WALLAC 1211 Rackbeta liquid scintillation counter. Heterotrophic prokaryote production, expressed as organic carbon produced, was calculated assuming a carbon to protein ratio of 0.86 and an isotope dilution factor of 2 [43]. Heterotrophic prokaryote production was converted to specific growth rate (µ; d^−1^) by dividing by heterotrophic biomass calculated assuming a carbon conversion factor of 12.4 fg C cell^−1^ [44].

### 2.4. Viral Mediated Mortality

Viral production was determined according to Winget et al. [45]. The aim of this method is to reduce the viral abundance in a sample to a level, which permits the accurate detection and enumeration of newly produced viruses while keeping the bacterial abundance at near ambient concentrations. At in situ temperature and under low light conditions, a 600 mL whole seawater sample was reduced to approximately 100 mL by recirculation over a 0.22 µm-pore-size polyether sulfone membrane (PES) tangential flow filter (Vivaflow 50; Sartorius stedim biotech, Göttingen, Germany) at a filtrate discharge rate of 40 mL min^−1^. Five hundred milliliters of virus-free water (generated by 30-kDa ultrafiltration Vivaflow 200, PES membrane; Sartorius stedim biotech, Göttingen, Germany) was then added. This reduction and resuspension procedure was repeated an additional two times. On the final iteration, the volume was reduced to approximately 50 mL and the filter was slowly back-flushed to obtain the remaining 50 mL volume in the system. The sample was then topped up with virus-free water (500 mL) and aliquoted into six 50 mL polycarbonate Greiner tubes. Triplicate samples were used to determine viral production due lytic infection, and Mytomycin C (Sigma-Aldrich; 1 µg mL^−1^ final concentration) was added the final three samples to determine the lysogenic induction rate from temperate phages. Fifty milliliter triplicate samples of untreated whole seawater and seawater filtered through 0.2 µm pore-sizes were also taken to provide estimates of net prokaryotic production and viral loss rates, respectively. One-milliliter subsamples for viral and prokaryotic abundance were taken at the start of the incubation (T_0_), after which the samples were incubated in darkness at in situ temperature and sub-sampled every 3 h for a total of 12–24 h. Samples were fixed, stored and enumerated as described previously.

Production rate of new viruses was determined from each replicate from the slope of a first-order regression of viral concentration over time. Prophage induction (VPC) was calculated as the difference between virus counts in unamended samples (lytic infection, VP) and those to which Mitomycin C was added. The in situ VP rate was corrected for potential prokaryote and virus loss due to sample processing and adsorption to sample tubes, respectively [45]. Samples showed no significant selective reduction of HNA and LNA cells after sample processing (i.e., two-way ANOVA: interaction between treatment and population *p* = 0.218). Estimates for daily virus-mediated mortality (VMM, expressed in cells L^−1^ d^−1^) were calculated by dividing lytic VP by a burst size of 20 [46].

### 2.5. Protozoan Mediated Mortality

Community grazing rates of prokaryotes were determined using fluorescently labeled natural bacteria (FLB) according to the procedure described by Sherr & Sherr [47]. Briefly, FLB (FLB stock contained 5 × 10^7^ mL^−1^, stored at −20 °C until use) were added to one-liter natural whole water samples (polycarbonate bottles) at approximately 10% of the natural concentration. Immediately after addition, a 20 mL subsample (T_0_) was taken and fixed with 10% glutaraldehyde (1% final concentration; EM-grade, Sigma-Aldrich, the Netherlands). The sample was then filtered onto a 0.2 µm pore-size black polycarbonate filter (25 mm, Whatman) and stored at −20 °C until analysis. The incubation bottles were closed such that no air was trapped inside, mounted on a slow rotating (0.5 rpm) plankton wheel, and incubated under in situ light and temperature. After 24 h incubation, a 20 mL subsample was taken and treated as previously described. The estimation of grazing rates (d^−1^) were determined as the natural log of the abundance of FLB in the T24 sample divided by the abundance of FLB in the T_0_ sample. Protozoan mediated mortality (PMM; L^−1^ d^−1^) was calculated as PMM = HPA_0_ – HPA_0_*e^rt^, where HPA_0_ is the abundance of HPA at the start of the incubation, and r equals the specific grazing rate (d^−1^) obtained from FLB experiments. 

### 2.6. Statistical Analysis

Statistical analysis was used to evaluate the potential of the VP method to selectively alter the relative abundance of HNA and LNA cells in the prokaryote community. Specifically, using the R statistical software [48] a two-way ANOVA was applied to all data to test for a significant interaction between treatment (i.e., VP and whole water) and subpopulation (i.e., LNA and HNA). A probability of α < 0.05 was to determine if the interaction was significant. 

To evaluate the hypotheses set forth in the introduction multivariate statistical analysis was applied to the data using the R statistical software supplemented by the vegan package [49]. Data exploration were carried out first according to Zuur et al. [50]. For H1, the response variables were HNA, LNA, the ratio of HNA to LNA cells (HNA:LNA), HPA, HPP and µ. Explanatory variables were latitude, MLD, Chl *a*, temperature, salinity, density, K_T_, O_2_, PhytoC, NH_4_, PO_4_, NO_2_, and NO_3_. In addition, depth layer was included as factor (i.e., single values per station/sample) to better discriminate how environmental conditions relate to changes in depth. PhytoC was log transformed and NH_4_ and NO_2_ log (x + 1) transformed to improve the homogeneity of variance and reduce the effect of outliers. Next, data were evaluated for collinearity of explanatory variables to obtain the most minimalistic model by calculating variance inflation factors using the R function corvif [51]. In a step-wise manner, all explanatory variables with variance inflation factors > 10 were removed from the model. For hypothesis H1, variance inflation factors analysis resulted in the selection of 7 continuous explanatory variables: latitude, MLD, K_T_, O_2_, NH_4_, NO_2_ and PhytoC and depth layer as a factor (levels: ML, MID, and DEEP). Additionally, LNA and HNA were removed as response variables due to their high correlation to HPA abundance (Pearson correlation: *n* = 32, *p*-value < 2.2 × 10^−16,^ r = 0.98 and r = 0.99, respectively). 

For H2, explanatory variables were temperature, K_T_, O_2_, NH_4_, PO_4_, NO_2_, NO_3_, HNA:LNA, HPA, HPP, µ, ratio of virus to heterotrophic prokaryote (VPR), and depth layer was included as a factor. Total heterotrophic prokaryote abundance, HNA:LNA and VPR were log transformed and NH_4_, NO_3_ and NO_2_ log (x + 1) transformed. Exploratory analysis resulted in the selection of 9 continuous explanatory variables for H2: temperature, K_T_, NH_4_, NO_2_, NO_3_, HNA:LNA, HPA, µ, VPR, and, depth layer. The response variables for H2 were VA, VP and VPC. For comparison, the same set of explanatory variables was used for H3. For this analysis, data exploration resulted in the selection of temperature, K_T_, NH_4_, NO_2_, HNA:LNA, HPA, HPP, µ, VPR, and depth layer.

Redundancy analysis (RDA) was applied to test H1-H3 using the three different datasets. RDA is a combination of multiple regression analysis and principal component analysis for multivariate data. Forward selection approach was used to select only explanatory variables that significantly contribute to the RDA model. Significance was assessed by a permutation test, using the multivariate pseudo-F as the test statistic [51]. A total of 9999 permutations were used to estimate *p*-values (α = 0.05) associated with the pseudo-F statistic. 

## 3. Results

### 3.1. Study Site

**Figure 2:**
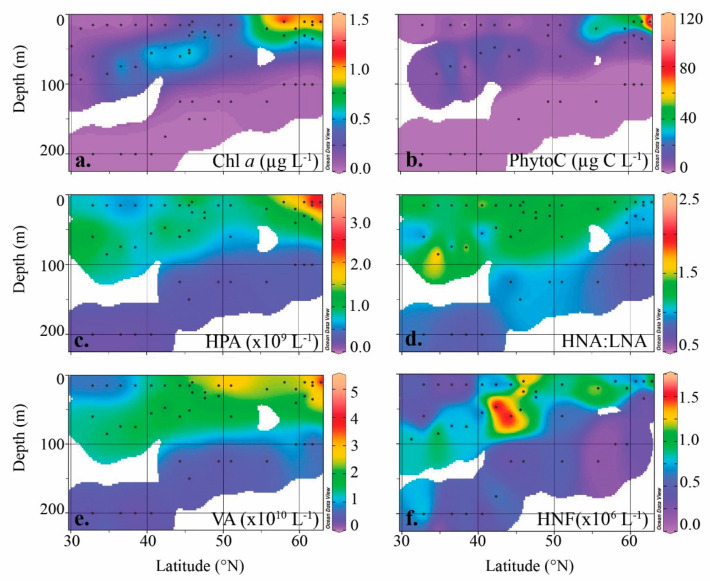
Biogeographical distributions of (**a**) Chl *a*, (**b**) phytoplankton carbon (PhytoC), (**c**) heterotrophic prokaryote abundance (HPA), (**d**) high nucleic acid fluorescent (HNA):low nucleic acid fluorescent (LNA), (**e**) virus abundance (VA), and (**f**) heterotrophic nanoflagellate abundance (HNF) across the Northeast Atlantic Ocean obtained during the STRATIPHYT cruise. Black dots indicate sampling points. Graphs were prepared with Ocean Data View version 5.2.

Temperature, salinity, and nutrients showed clear depth and latitudinal gradients (Supplement Table S1; [31]). In accordance with strong vertical stratification, the upper water column was characterized by low vertical mixing (K_T_), shallow MLDs (ranging from 19–44 m) and relatively high N^2^ (Table S1; [31]). The southern region (30–45 °N) was classified as oligotrophic based on ML concentrations of NO_3_ ≤ 0.13 µM and PO_4_ ≤ 0.03 µM [38], and Chl *a* ≤ 0.07 µg L^−1^ [52]. North of this region (i.e., 46–63 °N), inorganic nutrient concentrations within the ML increased to average 1.3 ± 0.7 µM NO_3_ and 0.13 ± 0.05 µM PO_4_, with highest concentrations north of 58 °N (stations 25–32) averaging 1.53 ± 0.43 µM and 0.15 ± 0.03 µM, respectively. Accordingly, Chl *a* and PhytoC concentrations in the surface ML increased along the meridional transect with subsurface maxima (i.e., DCM) present in stations of the oligotrophic region (Figure 2a,b). Specifically, average Chl *a* concentrations in the ML increased from 0.06 ± 0.02 µg L^−1^ in the oligotrophic region to 1.1 ± 0.2 µg L^−1^ north of 58 °N (Figure 2a). Similarly, PhytoC increased from 3.8 ± 2.4 µg C L^−1^ to 56.9 ± 37.5 µg C L^−1^ (Figure 2b). Inorganic nutrients in the DCM (i.e., MID depth samples) averaged 0.55 ± 0.72 µM NO_3_ and 0.06 ± 0.06 µM PO_4_, a marginal increase compared to the ML concentrations. In contrast, average Chl *a* and PhytoC concentrations in the DCM increased 7.5- and 2.6-fold compared to ML values (i.e., DCM 0.45 ± 0.25 µg L^−1^ and 10.0 ± 6.2 µg C L^−1^, respectively). At stations without a DCM, MID depth nutrient concentrations increased 4.8- and 3.5-fold compared to ML concentrations, averaging 5.47 ± 3.19 µM NO_3_ and 0.42 ± 0.19 µM PO_4_ and Chl *a* and PhytoC increased to 0.53 ± 0.28 µg L^−1^ and 35.21 ± 35.18 µg C L^−1^, respectively. Below 100 m, inorganic nutrients in the oligotrophic region averaged of 0.4 ± 0.1 µM and 7.2 ± 1.6 µM for PO_4_ and NO_3_, respectively. The northern region had similar nutrient concentrations in DEEP samples averaging 0.7 ± 0.1 µM and 11.1 ± 1.5 µM for PO_4_ and NO_3_. PhytoC and Chl *a* concentrations were low in DEEP samples and varied little over the latitudinal transect. Accordingly, PhytoC and Chl *a* in DEEP samples averaged 0.01 ± 0.01 µg L^−1^ and 0.02 ± 0.01 µg C L^−1^ in the southern oligotrophic region, and 0.02 ± 0.01 µg L^−1^ 0.21 ± 0.23 µg C L^−1^ in the north, respectively. 

### 3.2. Heterotrophic Prokaryotes

The abundance of the heterotrophic prokaryotes was consistently low in the surface mixed layer (ML) until 58 °N, with an average 6.9 ± 1.1 × 10^8^ prokaryotes L^−1^ in the oligotrophic region and increasing to an average of 17.6 ± 9.8 × 10^8^ L^−1^ in the north (Figure 2c). At MID depths, in the southern regions with a DCM, abundances were slightly higher compared to the ML averaging 9.6 ± 2.3 × 10^8^ prokaryotes L^−1^. Conversely, in the north, abundances were lower than ML values averaging 8.0 ± 2.8 × 10^8^ prokaryotes L^−1^. The HNA prokaryote population tended to numerically dominate at both the ML and MID depths (Figure 2d), comprising on average 55.5 ± 4.4 and 52.5 ± 9.4% of the total counts, respectively. The fraction of HNA varied little with latitude, with only a slight (i.e., ~2%) decrease between the southern and northern regions of the transect. Lowest heterotrophic prokaryote abundances were measured in DEEP samples with average concentrations ranging from 2.2 ± 0.3 × 10^8^ L^−1^ in the south to 2.9 ± 0.5 × 10^8^ L^−1^ in the north. The LNA population also had its greatest contribution to total prokaryotic abundance at this depth, comprising on average 54.0 ± 4.3% of the total counts (Figure 2d).

**Figure 3:**
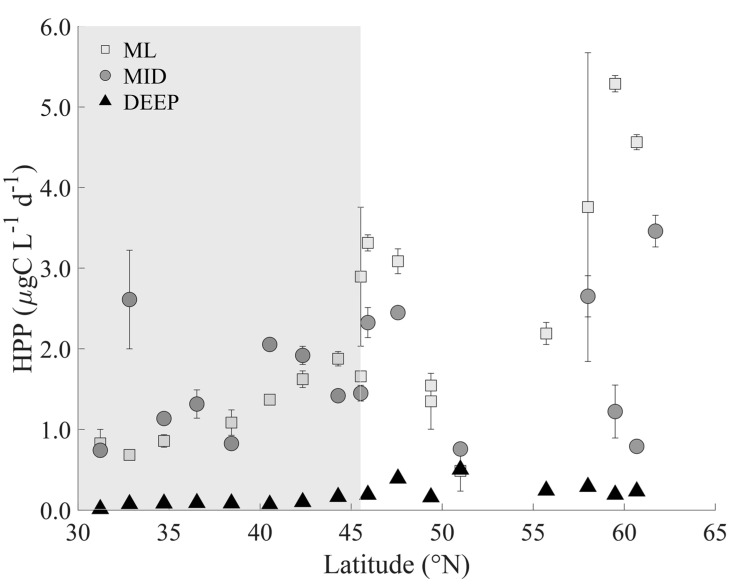
Heterotrophic prokaryote production (HPP) measured in the Northeast Atlantic during the summer STRATIPHYT cruise. Rates were obtained from 3 separate depths: the mixed layer (ML; 15 m), below the mixed layer (MID; 25–85 m), which included the deep-chlorophyll maximum where present (DCM; 47–85 m; defined by the presence of a subsurface peak in the vertical profile of Chl *a* autofluorescence), and deep (DEEP; 100–225 m). Error bars represent standard error (*n* = 3). The gray shaded area represented the latitudinal range of stations with a DCM present.

The heterotrophic prokaryote production in the ML increased steadily with latitude from 0.83 to 5.3 µg C L^−1^ d^−1^ (Figure 3). Similar to abundance, highest production rates were measured in the northern most stations (>58 °N) with an average production of 4.5 ± 0.8 µg C L^−1^ d^−1^. There was very little variation in production in ML and MID depth samples of the southern oligotrophic region (on average 1.4 ± 0.7 and 1.5 ± 0.6 µg C L^−1^ d^−1^, respectively). In the northern region, however, heterotrophic prokaryote production was on average slightly lower at MID depths (i.e., 2.0 ± 1.0 µg C L^−1^ d^−1^) compared to ML values (i.e., 2.8 ± 1.6 µg C L^−1^ d^−1^). Heterotrophic production was reduced in the DEEP samples to 0.09 ± 0.04 µg C L^−1^ d^−1^ in the south and to 0.28 ± 0.12 µg C L^−1^ d^−1^ in the north.

In order to ascertain key physicochemical parameters for the abundance and production of heterotrophic prokaryotes within our study, we applied a redundancy analysis (RDA) to our data. Forward selection revealed that only PhytoC and nitrate significantly contributed to the RDA model for H1 (Akaike Information Criterion (AIC): 21.74 and 18.82, F-statistic: 36.77 and 4.74, *p*-value: 0.005 and 0.010, respectively), explaining 56.9% and 4.5% of the variation in the data, respectively. Heterotrophic prokaryote abundance and activity at ML and MID depths was positively associated with PhytoC concentrations. Conversely, DEEP samples were characterized by low PhytoC and high nutrient concentrations and were associated with low prokaryote abundance, production, and cell specific growth rates, and a higher proportion of LNA cells. Oligotrophic stations (as indicated by the presence of a DCM) were characterized by lower nutrient and PhytoC concentrations, and were associated with higher HNA:LNA and lower prokaryote production and cell specific growth rates.

### 3.3. Viruses

Similar to heterotrophic prokaryote abundances, viral abundances were lowest in the ML of oligotrophic stations (average 11.0 ± 3.6 × 10^9^ L^−1^; Figure 2e) and increased at the oligotrophic boundary (~45 °N) to average 29.0 ± 9.5 × 10^9^ viruses L^−1^. The highest viral abundance of 52.6 × 10^9^ L^−1^ were measured in the ML at station 32. Viral abundances were slightly higher at MID depths compared to overlying ML at oligotrophic stations, i.e., 16.2 ± 4.2 × 10^9^ L^−1^ and increased only slightly in the northern region (18.3 ± 10.9 × 10^9^ L^−1^). Lowest abundances were measured in DEEP samples and varied little with latitude (increasing from 4.1 ± 1.2 in the south to 5.9 ± 3.1 × 10^9^ L^−1^ in the northern region). Average virus-to-prokaryote ratio (VPR) varied little between ML and MID depths but both showed an increase between the southern region (16.2 ± 4.8 and 17.5 ± 5.1) and northern region of the transect (22.0 ± 14.2 and 21.7 ± 6.8, respectively). Conversely, DEEP samples showed little variation in VPR between the two regions, i.e., 19.1 ± 6.4 and 20.3 ± 9.5, respectively. The V1 virus group dominated the viral community, comprising on average 68.7 ± 8.0% of the total counts. The contribution V1 to total virus abundance increased between the south and northern regions, with greatest differences measured in the ML and MID depths (ML: 62 ± 5 to 75 ± 4%, MID: 65 ± 7 to 71 ± 11% and DEEP: 69 ± 7 to 71 ± 10%).

**Figure 4:**
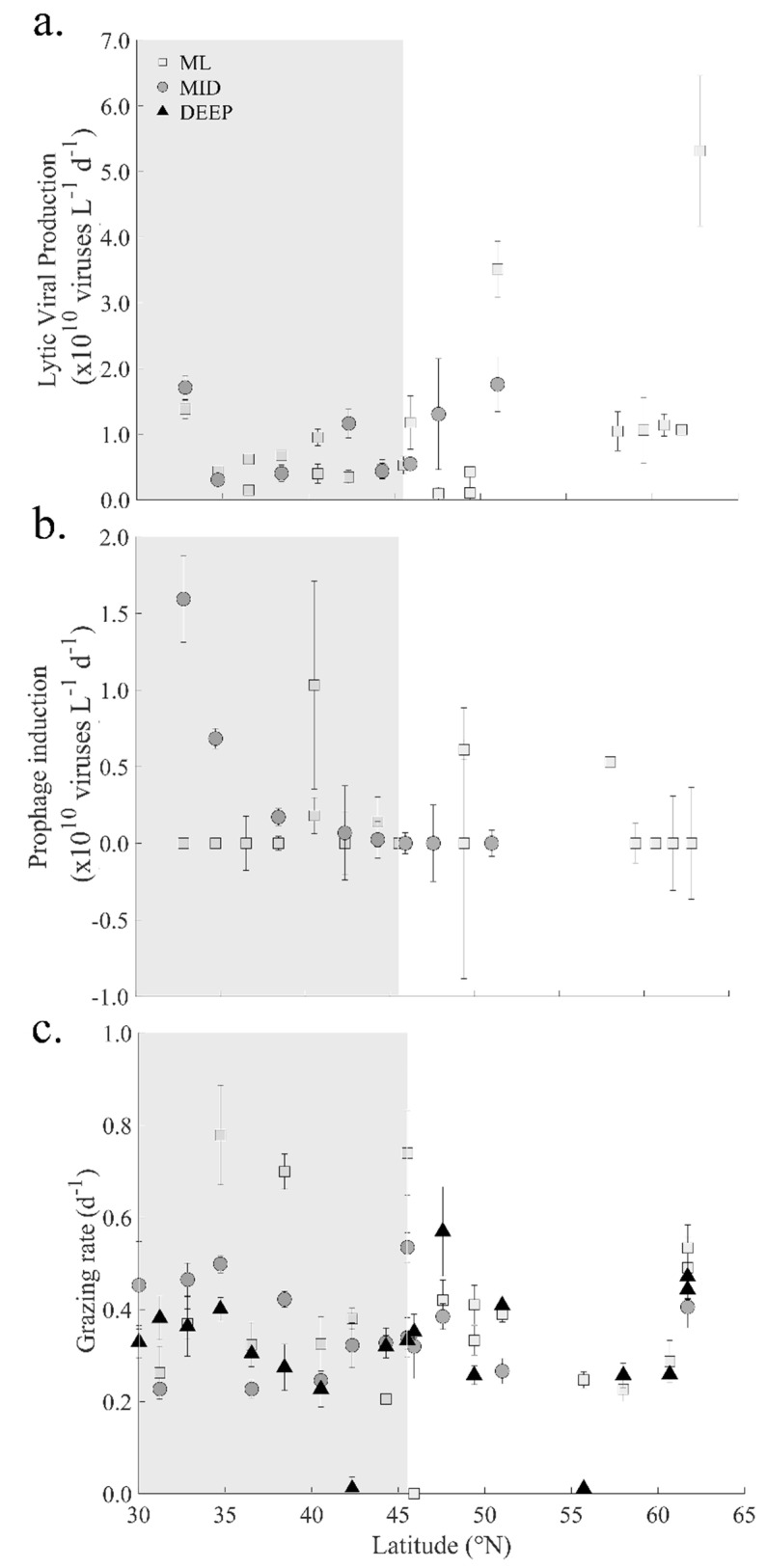
Average rates of viral and grazing activity within prokaryotes communities in the Northeast Atlantic during the summer STRATIPHYT cruise. Daily rates of (**a**) lytic viral production (VP), (**b**) Mitomycin C prophage induction (VPC), and (**c**) microzooplankton grazing. Rates were obtained from three separate depths: the mixed layer (ML; 15 m), below the mixed layer (MID; 25–85 m), which included the deep-chlorophyll maximum where present (DCM; 47–85 m; defined by the presence of a subsurface peak in the vertical profile of Chl *a* autofluorescence), and deep (DEEP; 100–225 m). Error bars represent standard error (*n* = 3). Gray shaded area represented the latitudinal range of stations with a DCM present.

Rates of total lytic virus production at ML and MID depths were largely a reflection of the production of viruses of the V1 group (i.e., 94.3 ± 19.4%). In the ML, lytic virus production increased from 0.6 ± 0.4 × 10^10^ viruses L^−1^ d^−1^ in the oligotrophic south to 1.5 ± 1.7 × 10^10^ viruses L^−1^ d^−1^ in the north (Figure 4a), resulting in a greater than 2-fold increase in virus-mediated mortality from 3.0 ± 1.7 to 7.5 ± 8.3 × 10^8^ cell lysed L^−1^ d^−1^. The prophage induction rates in ML samples of the south were about a third of the lytic production rates, averaging 0.2 ± 0.3 × 10^10^ L^−1^ d^−1^ (Figure 4b). Measurable rates of prophage induction in the north were relatively low (compared to lytic production) and detected at only 4 stations (i.e., 16, 19, 25 and 30–2), averaging 0.1 ± 0.2 × 10^10^ viruses L^−1^ d^−1^. In the MID depth samples of the south, lytic virus production rates were comparable to ML values, i.e., 0.8 ± 0.6 × 10^10^ viruses L^−1^ d^−1^ or 3.0 × 10^8^ cell lysed L^−1^ d^−1^. Although viral production was only determined at MID depths at 3 stations in the north (16, 18, 21), rates increased from 0.5 × 10^10^ to 1.8 × 10^10^ L^−1^ d^−1^ corresponding to a viral mediated morality ranging from 2.7 to 8.8 × 10^8^ cell lysed L^−1^ d^−1^. The highest rates of prophage induction were recorded in the MID depth samples from the DCM of the oligotrophic region which averaged 0.5 ± 0.7 × 10^10^ viruses produced L^−1^ d^−1^. Moreover, the prophage induction rates within these samples declined hyperbolically with latitude (Figure 4b).

**Figure 5:**
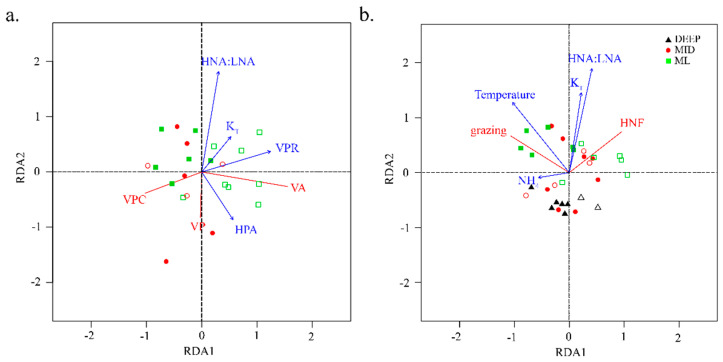
Redundancy analysis (RDA) correlation triplots of factors important in structuring the abundance and activity of mortality agents of (**a**) viruses and (**b**) heterotrophic nanoflagellates during STRATIPHYT. Response variables are shown in red and explanatory variables in blue. Symbols represent individual sampling points (a:*n* = 22 and b:*n* = 32) and illustrate from what depth layer samples originated; shape and color coded according to the depth layer and filled according to the presence or absence of a deep-chlorophyll maximum (closed = present and open = absent). The total variance explained by the RDA models in panel a and b were 58.8% and 38.9%, respectively. Abbreviations represent viral abundance (VA), lytic viral production (VP), Mitomycin C prophage induction (VPC), virus-to-prokaryote ratio (VPR), heterotrophic prokaryote abundance (HPA), nitrate (NO_3_), HNA to LNA ratio (HNA:LNA), ammonium (NH_4_), vertical mixing coefficient (K_T_), and heterotrophic nanoflagellate abundance (HNF).

Figure 5a illustrates the most parsimonious RDA model for hypothesis H2. Forward selection revealed that VPR, HPA, HNA:LNA and K_T_ all significantly contributed to the RDA model at an α of 0.05. The first two axes of the RDA triplot (Figure 5a) were driven by VPR and HNA:LNA and explained 43.7% and 11.3% of the variation in the data, respectively. Lytic virus production was inversely associated to HNA:LNA and K_T_ and positively to heterotrophic prokaryote abundance (HPA). Conversely, stations with low VPR, viral abundance, and K_T_ were associated with higher prophage induction rates. Moreover, viral abundance and lytic production were not strongly coupled. Stations outside the oligotrophic region (i.e., no DCM; right quadrants) were characterized by higher abundances of heterotrophic prokaryotes and viruses, and thus were also associated with higher VPR.

### 3.4. Heterotrophic Protists 

The abundance of HNF in the ML increased nearly 2-fold from south to north, i.e., 5.1 ± 2.1 to 9.6 ± 2.6 × 10^5^ L^−1^ (Figure 2f). At oligotrophic sites, HNF abundances at MID depths were on average about 2-fold higher compared to ML values (i.e., 9.6 ± 4.1 × 10^5^ HNF L^−1^). In the north, however, the abundances of HNF were reduced at MID depths compared to the ML (i.e., 7.8 ± 5.2 × 10^5^ L^−1^). Lowest HNF abundances were measured in DEEP samples, decreasing slightly from 4.9 ± 1.9 × 10^5^ L^−1^ in the south to 3.4 ± 1.7 × 10^5^ L^−1^ at northern stations. Despite lower abundances of HNF, the specific community grazing rates were on average higher at oligotrophic stations compared to those measured at stations in the north (averaging 0.45 ± 0.22 and 0.33 ± 0.15 d^−1^, respectively). When extrapolated to protist mediated mortality, rates increased from 2.3 ± 8.6 × 10^8^ in the south to 4.9 ± 4.2 × 10^8^ cells grazed L^−1^ d^−1^ in the north. Samples from MID depths showed very little variation in measured community grazing rates and protist mediated mortality between the two regions (i.e., 0.37 ± 0.11 d^−1^ compared to 0.34 ± 0.06 d^−1^ and 3.0 ± 1.1 × 10^8^ cells grazed L^−1^ d^−1^ compared to 2.6 ± 1.3 × 10^8^ cells grazed L^−1^ d^−1^, respectively). Community grazing rates in DEEP samples were still relatively high (0.29 ± 0.11 d^−1^ in the south and 0.34 ± 0.16 d^−1^ in the north) compared to HNF abundances measured at those depths (Figure 4c). Protist mediated mortality in DEEP samples increased slightly from 0.5 ± 0.2 × 10^8^ cells grazed L^−1^ d^−1^ within the oligotrophic region to 0.7 ± 0.5 × 10^8^ cells grazed L^−1^ d^−1^ in the north. 

Figure 5b illustrates the RDA model for hypothesis H3. Forward selection revealed that temperature, HNA:LNA, and K_T_ significantly contributed to the RDA model at an α of 0.05. Ammonium was slightly below (*p* = 0.055) the set alpha, however, it was retained in the final model as it significantly contributed to the constrained portion of the variance. Nevertheless, a large proportion (53%) of unconstrained variation remained (i.e., variation in response variables that is non-redundant with the variation in response variables). The first two axes of the RDA triplot explained 26.2% and 12.8% of the variation in the data, respectively. Temperature (negative direction) was the main variable contributing to the formation of the first axis, while the second axis was driven by HNA:LNA and K_T_. Stations characterized by increased K_T_, higher HNA:LNA and lower NH_4_ were associated with higher HNF abundances. Increased community grazing grates were associated with higher temperature environments. Interestingly, HNF abundance was not correlated to either temperature or community grazing rate (i.e., in a RDA plot correlations are reflected in the angles between lines, wherein a 90° angle represents no correlation).

### 3.5. Heterotrophic Prokaryote Mortality

Averaged over the ML and MID depths, total mortality increased from 6.4 ± 2.9 in the oligotrophic southern region to 9.3 ± 5.5 cells L^−1^ d^−1^ in the north. Specifically, total mortality in the ML increased from an average of 5.8 ± 1.9 to 9.8 ± 6.3 × 10^8^ cells L^−1^ d^−1^, and at MID depths increased from 7.3 ± 4.0 × 10^8^ cells L^−1^ d^−1^ to 8.0 ± 3.2 × 10^8^ cells L^−1^ d^−1^. In general, prokaryotic losses were dominated by virus mediated mortality (Table 1).

**Figure 6:**
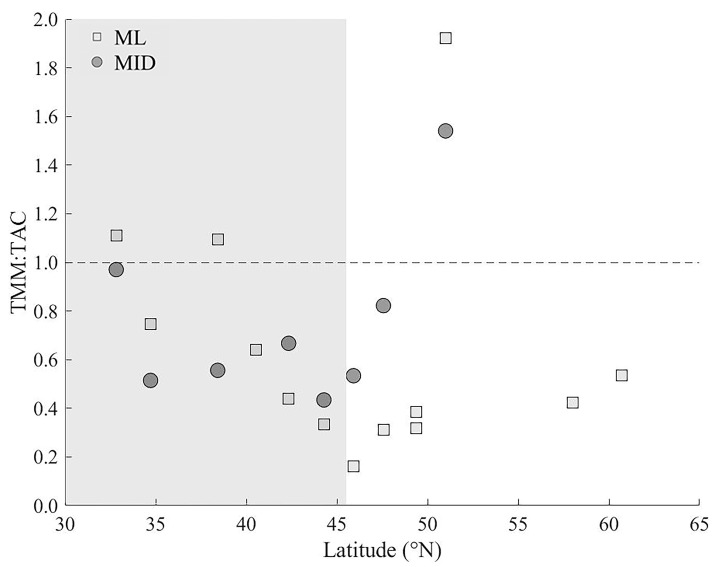
The ratio of total microbial mediated mortality (TMM; VMM + PMM) to total available prokaryotic carbon (TAC; standing stock biomass + production) as a function of latitude for the ML and MID depth samples. The gray shaded area represented the latitudinal range of stations with a DCM present.

The ratio of total microbial-mediated mortality (TMM; VMM + PMM) to total available prokaryotic carbon (TAC; standing stock biomass + production) in the ML and MID samples showed a gradual decrease in oligotrophic region from around 1.0 to 0.4 (Figure 6). North of 45 °N, the ML increased from around 0.2 to 0.4 before reaching a maximum of 1.9 at 51 °N that was the closest near-shore station sampled (Station 21). In the northern most region (>58 °N), TMM:TAC averaged 0.5. The MID depth showed a similar trend, increasing north of 45 °N from 0.5 to 0.8 before reaching also reaching a maximum of 1.5 at station 21.

## 4. Discussion

### 4.1. Heterotrophic Prokaryote Abundance and Activity

Our study area in the North Atlantic Ocean offered a large-scale gradient from permanently stratified subtropics to the seasonal stratified temperate region [31,53]. Heterotrophic prokaryote abundance, production, and cell specific growth were all tightly coupled to Chl *a* and phytoplankton carbon concentrations. Vertical stratification was found to play an important role in regulating the size and composition of phytoplankton communities in this area during the time of our study [31]. The results presented here suggest that these alterations in phytoplankton biomass have direct consequences for heterotrophic prokaryotes. Phytoplankton and heterotrophic prokaryotes are inherently linked by their opposing roles as the primary producers and consumers of dissolved organic matter, respectively, and consequently co-vary across a wide range of aquatic ecosystems [54,55,56]. A strong correlation between heterotrophic prokaryote production and specific growth rates also indicates that the variability in specific growth rate (i.e., production/biomass) was an important mechanism governing prokaryote production. The availability of dissolved organic carbon is considered as the primary factor regulating heterotrophic prokaryote activity in marine systems [57,58,59]. For our data, phytoplankton carbon alone explained more than half (~57%) of the variability in heterotrophic prokaryotic community dynamics. Accordingly, food web processing (i.e., dissolved organic matter release, sloppy feeding, viral lysis, etc.) likely provided a vital source of available DOC for the heterotrophic prokaryotic community. 

The HNA cells are typical (but not always; [10,13,60]) considered to be the more active subpopulation within a heterotrophic prokaryote community and are often associated with higher cell-specific activity rates compared to LNA cells [10,61]. Indeed, our results reveal a positive association of HNA:LNA with prokaryotic production and specific growth rate, suggesting a higher metabolic activity of HNA cells [1,62,63]. Accordingly, the proportion of HNA cells in a community is generally expected to be positively associated with ecosystem productivity. However, our results reveal HNA cells as a predominant component of the prokaryote community of the southern oligotrophic subtropical region. Higher percentages of HNA cells have been reported for the DCM, but in general the proportion of LNA cells is typically been found to increase with oligotrophy [7,8,64]. Moreover, there was a discernible decrease in HNA:LNA in the surface waters north of 58° where productivity was maximal. At the time of this study, phytoplankton mortality at low and mid latitudes was dominated by viral mortality, shifting to a grazing dominated system at higher latitudes (>56 °N) [42]. Phytoplankton mortality has substantial effects on the production and composition of DOM, and consequently on the activity and composition of the surrounding heterotrophic prokaryote community [20,21,65,66,67]. In order to examine the relationship between phytoplankton mortality mode (i.e., virus versus grazing) previously reported [42] and the trends in HNA:LNA of the current study, we combined data for all stations and depths where simultaneous measurements were acquired. For optimal comparison of the data, the ratio of viral lysis to grazing rate of phytoplankton populations were averaged across all groups with maxima capped at 5.5. Indeed, we found a significant positive correlation between phytoplankton mortality mode and HNA:LNA (Pearson *r* = 0.6, *n* = 14, *p*-value = 0.005). Accordingly, phytoplankton mortality processes likely played a major role in the distribution of heterotrophic prokaryote subpopulations during our study. 

The HNA prokaryotes often have a strong phylogenetic association with copiotrophic members of Bacteroidetes, Gammaproteobacteria, and Aphaproteobacteria [1,68]. A common characteristic of many of these copiotrophic strains is their propensity to respond to and utilize proteins, peptides, and complex polysaccharides, particularly those associated with phytoplankton blooms [69,70,71]. This may provide them with a selective advantage that enables them to successfully exploit transient nutrient bursts, like those associated with the cell leakage and lysis due to viral infection. Indeed, viral infection and lysis of phytoplankton has been linked to enrichment of HNA cells and associated taxa (i.e., Gammaproteobacteria and Aphaproteobacteria) in marine prokaryotic communities [20,67]. The inverse association between HNA:LNA and nitrate, which has also been reported previously for the Atlantic [8,72], may then be indicative of a reduced reliance of HNA cells on inorganic sources of nitrogen (cellular material released due to lysis is rich in organic phosphorus and nitrogen compounds).

### 4.2. Viral Mediated Mortality of Heterotrophic Prokaryotes

Viral proliferation is dependent upon its hosts, consequently virus abundance often co-varies with their numerically dominate hosts—the heterotrophic prokaryotes [73,74,75]. Accordingly, viral abundance and the fraction of V1 viruses in our community were strongly associated with ecosystem productivity. In addition to host availability, the rate at which viruses encounter a heterotrophic prokaryote cell (e.g., assumed to be proportional to VPR), and the subpopulation most likely to encounter (e.g., HNA:LNA) were also important in regulating viral dynamics. Rates of viral production can be affected by host physiology, either through host growth rate or prophage induction [76,77,78], however, specific prokaryote growth rate did not appear to significantly influence the variability in the abundance or activity within our viral community. Instead, lytic virus production was inversely associated with the fraction of HNA cells in the community, and was uncoupled from viral abundance. Data from several studies suggest a link between HNA cells and the dominate (both in terms of abundance and contributions to total lytic production) virus group in the present study—V1 [30,79,80]. This inverse relationship between lytic virus production and the fraction of HNA cells in the community may, therefore, reflect predator-prey oscillations in virus production with the density of active hosts. 

The prevalence of the different viral replication modes (i.e., lytic versus lysogenic) can be related to the trophic status of a system [81,82,83]. In the DCM, prophage induction decreased hyperbolically with latitude in a manner consistent with increases in nutrient and Chl *a* concentrations (Pearson correlation to PO_4_: r = −0.82, *p*-value = 0.01 and Chl *a*: r = −0.72, *p*-value = 0.04). This suggests that trophic status was likely an important factor modulating lysogeny in our bacteriophage the community in the DCM [76,77]. Conversely, prophage induction was absent or low in the surface ML of the subtropical region (south of 38 °*n*), with the highest frequency of occurrence in the transition zone (38–46 °*n*) between the strongly stratified oligotrophic region and the less stratified northern region. One possible explanation is that prolonged stratification in the subtropical region may have subjected hosts to high levels of solar radiation (particularly UV), which can act as an inducing agent and reduce the yield of viral production from Mitomycin C treatment [84,85]. Overall, prophage induction appeared to be strongly tied to low VPR, indicated that the rate at which viruses encountered a heterotrophic prokaryote cell was the primary factor governing lysogeny. This aligns well with the theory that the lysogeny represents a survival strategy of viruses to endure periods of low host abundance or production [81,86].

### 4.3. Controls on Grazing Mortality

The densities of heterotrophic prokaryotes and HNF are thought to be strongly related to the degree of eutrophication with a predictable numerical relationship of prokaryotic to HNF abundances (HPA:HNF) among oligotrophic and eutrophic systems [87,88]. Indeed, HNF increased in proportion to the prokaryotic abundance with HPA:HNF averaging 1.1 ± 0.8 × 10^3^ in the subtropical region (<48 °*n*) and increasing to 1.7 ± 1.2 × 10^3^ in the less stratified northern region. Temperature and turbulence were the primary physicochemical factors regulating the variability in the abundance and activity of bacterivorous protozoans across our latitudinal gradient. Temperature [33,89] and turbulence [34,35] have both been shown to have a positive effect on growth and grazing rates of bactivorous protists. 

Heterotrophic nanoflagellate abundance was decoupled from measured grazing rates (i.e., in a RDA plot correlations are reflected in the angles between lines, wherein a 90° angle represents no correlation), and was inversely correlated to ammonium concentrations. This may indicate strong top-down control of protists [90,91] and subsequent regenerated nitrogen (particularly in the oligotrophic subtropical region [92,93]), and/or heterotrophic nanoflagellates were not responsible for the majority of the bactivory in our heterotrophic prokaryotes community [47,94]. In addition to flagellated protists, pelagic ciliates are an important source of bacterial mortality [95]. However, the growth and feeding activity of ciliates are more sensitive to turbulence than their flagellate counterparts and have been shown to be negatively affected by increase in turbulence [35,36]. In order to better understand the decoupling of HNF and grazing rates (absence of a strong Lotka–Volterra predatory–prey relationship in between HNA:LNA and HNF), we applied the qualitative model proposed by Gasol [96], which provides a framework for evaluating the strength of top-down and bottom-up factors controlling HNF. All of our data fall well below the mean realized abundance (MRA) line supporting the theory that decoupling was due to strong top-down control of HNF (Figure S1). 

Bacterivorous protozoa are thought to preferentially select for the actively growing cells of the bacterial assemblage by size-selectively grazing larger and more active cells in the community [24,97]. Our results indicate that grazing rate and HNF abundance increased with fraction of the HNA cells in the heterotrophic prokaryote community, supporting evidence that physiological and ecological differences in LNA and HNA cells may impact marine food webs by protozoans selectively grazing on HNA cells [9,98]. In addition, K_T_ values were also strongly related to HNA:LNA, suggesting that stratification may have also influenced the availability of prey type. 

### 4.4. Ecosystem Dynamics

Our results reveal strong bottom-up control (resource availability) of heterotrophic prokaryote activity and as such variations in phytoplankton carbon (driven by vertical stratification) has direct consequences for the abundance and activity of the heterotrophic prokaryote community. The production and availability of DOM can vary across phytoplankton species, their growth phase, as well as the type of nutrients that limit growth [99,100,101], all of which are likely influenced by vertical stratification. The significant relationship between prokaryotic HNA:LNA and the phytoplankton viral lysis to grazing ratio implies that viral activity within the surrounding phytoplankton community provided HNA a (likely transient) opportunity to outcompete LNA cells which typically prevail under more oligotrophic conditions [7,8]. These results support evidence that organic matter released from algal cells by viral lysis not only provides substrate for surrounding heterotrophic prokaryotes, but is important in structuring community composition [20,67]. HNA:LNA was an important factor regulating the mortality of heterotrophic prokaryotes and thus the impact of these alterations likely propagated throughout the ecosystem. 

In order to disentangle the role of viruses and heterotrophic nanoflagellates in controlling heterotrophic prokaryote communities we compared covariations in the abundances of these two mortality agents in the ML and MID sample depth (Figure S2). Viral abundance (Pearson *r* = 0.84, *n* = 16, *p*-value = 1.9 × 10^−8^) and lytic virus production (*r* = 0.46, *n* = 16, *p*-value = 0.02) were positively associated with HNF in the ML. However, no connection was found between grazing rates and viral abundance, prokaryotic abundance, or virus mediated mortality. In the MID we found an opposite effect of HNF on viral abundance (*r* = −0.39, *n* = 12, *p*-value = 0.09) and lytic virus production (*r* = −0.57, *n* = 8, *p*-value = 0.04), supporting evidence that the presence of HNF can reduce viral activity [80,83]. As viruses and HNF compete for the same resource (i.e., prey/host), exploitative competition is expected, that is, the activity of one reduces the resource and thereby the activity of the other (Figure S2c). In addition to competition, HNF may also reduce the abundance and production of viruses by direct feeding, grazing on infection sensitive hosts (favoring species less susceptible to viral infection; [102]), or by predation on infected cells. Direct predation on viruses by HNF occurs at much lower rates compared heterotrophic prokaryote prey (4%), resulting in negligible contributions to the removal of viruses (around 0.1% of the virus community h^−1^) [103,104].

Overall, viral-mediate morality was the primary top-down process regulating the heterotrophic prokaryotic communities in the current study, which may be attributed to the strong top down regulation of HNF [105,106]. Viruses were responsible for an average of 55 ± 22% (ranging from 12 to 100%, median 60.3) of the total mortality occurring within the heterotrophic prokaryote communities. This agrees well with the literature that generally attributes 10–50% of the total bacterial mortality in the surface ocean to viruses [102,107]. In terms of carbon flux, our data suggest that around 39% of the total available carbon (i.e., standing stock + production) of our prokaryote communities was cycled back into the water column by viral activity compared to 26% entering the food web by grazing. Moreover, the ratio of total mortality and available prokaryote carbon reveals that over the latitudinal gradient in stratification the heterotrophic prokaryote community gradually moves from a near steady state system regulated by high turnover in subtropical region to net heterotrophic production in the temperate region. This supports evidence that loss within heterotrophic prokaryotic communities expressed as a fraction of either biomass or production tends to be negatively associated with ecosystem productivity [108,109,110,111,112].

## Figures and Tables

**Table 1:** Summary of heterotrophic prokaryote mortality rates. Data for total microbial-mediated mortality (TMM), viral-mediated mortality (VMM) and protist-mediated mortality (PMM) are presented as averages (×10^8^ cells L^−1^ d^−1^) ± standard deviation for the mixed layer (ML; 15 m) and mid sampling depths (MID; 25–85 m) of the southern (S; 30–45° N) and northern (N; 46–63° N) regions of the meridional transect.

	ML		MID	
	S	N	S	N
VMM	3.0 ± 1.8	5.4 ± 5.1	4.0 ± 3.1	6.0 ± 3.1
PMM	2.3 ± 0.9	4.9 ± 4.2	3.0 ± 1.1	2.6 ± 1.3
TMM	5.8 ± 1.9	9.8 ± 6.3	7.3 ± 4.0	8.0 ± 3.2

## Supplementary Materials

The following are available online at https://www.mdpi.com/1999-4915/12/11/1293/s1, Figure S1: evaluating the strength of top-down and bottom-up factors controlling HNF, Figure S2: interactions of between viruses and heterotrophic nanoflagellates. Table S1: physicochemical data used for multivariate analysis.
