# Supplementary material for: Significance of Viral Activity for Regulating Heterotrophic Prokaryote Community Dynamics along a Meridional Gradient of Stratification in the Northeast Atlantic Ocean
Source: Viruses. 2020 Nov 12;12(11):1293. doi: 10.3390/v12111293 (PMC7696675; doi:10.3390/v12111293)
Supplement: Supplementary file 1 [file viruses-12-01293-s001.pdf]

Significance of Viral Activity for Regulating Heterotrophic Prokaryote Community Dynamics along a Meridional Gradient of Stratification in the Northeast Atlantic Ocean

Kristina D. A. Mojica [1,2,*] and Corina P. D. Brussaard [1]

[1] Department of Biological Oceanography, Royal Netherlands Institute for Sea Research (NIOZ), P.O. Box 59, 1790 AB Den Burg, Texel, The Netherlands; Corina.Brussaard@nioz.nl

[2] Division of Marine Science, School of Ocean Science and Engineering, The University of Southern Mississippi, Stennis Space Center, 39529, USA

* Correspondence: Kristina.Mojica@usm.edu; Tel.: +1-22-8688-3003

Table S1. Station list with physiochemical characteristics, Chl *a* and phytoplankton carbon (PhytoC) concentration for the depths layers sampled, i.e., mixed layer (ML), MID (* indicates the presence of a DCM), and DEEP samples (100-225 m).

Station	Latitude (°N)	Longitude (°E)	Depth (m)	Depth Layer	MLD (m)	Temperature (°C)	Salinity	K_T ($m^2 s^{-1}$)	NO_3 (µM)	PO_4 (µM)	NH_4 (µM)
1	30.014	-15.069	87	MID*	30	17.74	36.60	-4.51	0.05	0.03	0.05
			225	DEEP		15.74	36.28	-7.91	5.76	0.35	0.06
2	31.221	-14.866	20	ML	34	22.82	36.81	-0.98	0.05	0.00	0.06
			93	MID*		17.90	36.64	-5.17	0.06	0.00	0.03
			225	DEEP		16.43	36.40	-7.91	4.37	0.23	0.02
3	32.825	-14.589	15	ML	25	22.78	36.88	-1.82	0.05	0.00	0.08
			60	MID*		18.05	36.55	-4.70	0.07	0.00	0.06
			200	DEEP		15.64	36.26	-7.91	5.24	0.28	0.06
5	34.720	-14.258	15	ML	29	22.27	36.74	-2.45	0.00	0.01	0.10
			85	MID*		16.13	36.27	-5.05	0.00	0.03	0.16
			225	DEEP		14.02	35.97	-7.91	7.53	0.45	0.06
7	36.526	-13.934	15	ML	22	20.63	36.24	-3.42	0.00	0.00	0.06
			74	MID*		16.13	36.25	-5.62	0.14	0.02	0.06
			200	DEEP		13.89	35.97	-7.91	7.37	0.44	0.04
7-2	36.526	-13.934	15	ML	22	20.60	36.25	-3.43	0.06	0.03	0.07
9	38.424	-13.586	15	ML	40	21.06	36.39	-3.15	0.04	0.02	0.06
			75	MID*		14.90	36.07	-5.15	1.32	0.13	0.18
			200	DEEP		13.22	35.86	-7.91	9.37	0.56	0.04
11	40.528	-13.191	15	ML	24	19.78	35.96	-3.13	0.00	0.01	0.06
			55	MID*		15.22	35.96	-5.12	0.00	0.04	0.07
			200	DEEP		13.31	35.87	-7.91	6.89	0.42	0.05
11-2	40.528	-13.191	15	ML	24	19.85	35.99	-3.12	0.00	0.01	0.05
13	42.337	-12.884	15	ML	26	18.75	35.80	-2.98	0.05	0.03	0.00
			47	MID*		14.67	35.82	-4.79	0.09	0.07	0.00
			175	DEEP		12.42	35.71	-7.91	8.91	0.54	0.03
15	44.283	-12.605	15	ML	25	18.35	35.81	-2.61	0.05	0.03	0.06
			60	MID*		14.35	35.79	-4.66	2.05	0.17	0.07
			125	DEEP		12.74	35.78	-7.91	7.93	0.48	0.06
16	45.917	-12.363	10	ML	21	16.88	35.65	-2.71	0.10	0.04	0.04
			25	MID		16.15	35.65	-3.43	1.17	0.13	0.06
			125	DEEP		12.34	35.63	-7.91	8.94	0.54	0.06

17	45.527	-12.426	15	ML	39	17.83	35.76	-3.01	0.06	0.03	0.08
			30	ML		17.84	35.76	-3.99	0.11	0.05	0.07
			51	MID*		14.16	35.72	-5.02	1.04	0.10	0.11
			150	DEEP		12.17	35.69	-7.91	8.40	0.53	0.05
17-2	45.526	-12.426	15	ML	39	17.89	35.77	-2.99	0.05	0.02	0.04
18	47.569	-12.110	25	ML	29	16.60	35.66	-3.08	0.07	0.05	0.11
			33	MID		15.29	35.66	-3.94	5.67	0.37	0.12
			150	DEEP		12.04	35.58	-7.91	9.34	0.59	0.10
19	49.382	-11.829	15	ML	44	15.81	35.52	-2.56	1.15	0.12	0.31
			30	ML		15.75	35.52	-3.67	1.29	0.16	0.39
			125	DEEP		11.44	35.55	-7.91	10.05	0.63	0.06
21	51.000	-11.567	15	ML	35	15.90	35.52	-2.48	1.15	0.15	0.39
			60	MID		12.39	35.57	-4.68	5.83	0.44	0.57
			125	DEEP		11.55	35.58	-7.91	9.89	0.63	0.07
24	55.713	-14.278	20	ML	27	13.89	35.34	-3.19	2.58	0.19	0.07
			125	DEEP		11.09	35.47	-7.91	10.70	0.65	0.00
25	58.002	-16.516	10	ML	19	13.47	35.34	-2.81	1.18	0.11	0.09
			30	MID		12.25	35.36	-4.04	5.57	0.40	0.29
			100	DEEP		9.84	35.40	-5.35	12.30	0.78	0.07
27	59.499	-18.067	20	ML	24	13.95	35.22	-3.81	2.08	0.18	0.19
			40	MID		11.71	35.25	-5.02	5.67	0.52	1.94
			100	DEEP		9.39	35.27	-5.35	12.49	0.79	0.03
29	60.684	-19.339	10	ML	22	13.07	35.26	-2.78	2.00	0.19	0.17
			30	MID		9.83	35.23	-3.84	11.46	0.74	0.05
			100	DEEP		9.48	35.33	-4.84	12.67	0.79	0.01
30	61.712	-20.485	10	ML	26	13.15	35.20	-3.13	1.00	0.14	0.30
			35	MID		12.45	35.20	-4.35	2.91	0.31	0.92
			100	DEEP		9.14	35.28	-4.80	12.68	0.83	0.05
30-2	61.715	-20.489	15	ML	26	13.12	35.23	-3.33	1.38	0.15	0.33
32	62.800	-21.736	10	ML	22	12.77	35.28	-4.09	1.52	0.14	0.64

Data for K_T and nutrients were originated from (Jurado et al. 2012b) and (Mojica et al. 2015), respectively.

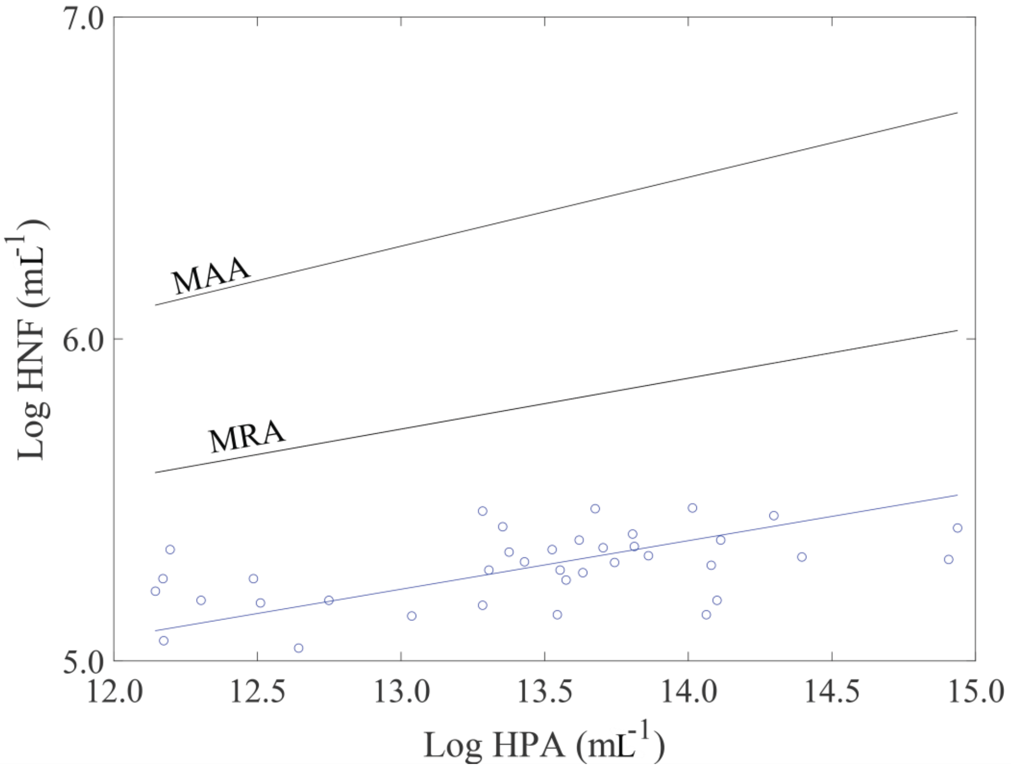

FIG. S1. Observations of the abundance of heterotrophic prokaryotes (HPA) and heterotrophic nanoflagellates (HNF) for all stations and depths of STRATIPHYT I and their relationship to the maximum attainable abundance (MAA) and mean realized abundance (MRA) lines [98].

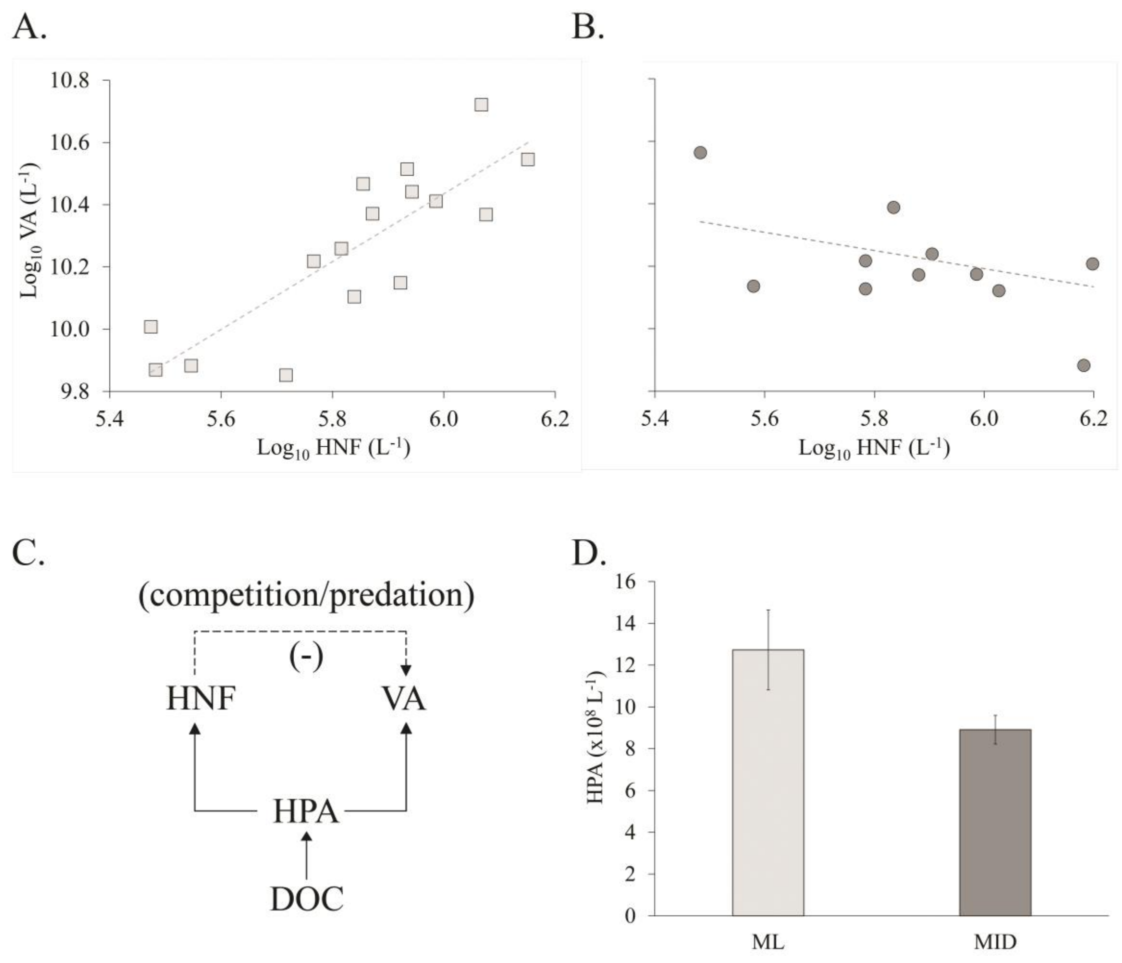

FIG. S2. The relationship between the abundance of viruses (VA) and heterotrophic nanoflagellates (HNF) for **(A)** ML and **(B)** MID depths. **(C)** A conceptual model illustrating the potential for HNA to negatively affect VA through competition or predation. **(D)** A bar graph demonstrating that the negative relationship in **(B)** is likely driven by competition drive by a significantly lower HPA in the MID depth samples (type III t-test; *p*-value = 0.04).
